# Single Nucleotide Polymorphisms in IFN-*γ* Signaling Pathway Associated with Risk of Hepatitis B Virus Infection in Chinese Children

**DOI:** 10.1155/2020/8121659

**Published:** 2020-01-22

**Authors:** Yang Zhuo, Yalan Yang, Mingjun Zhang, Ying Xu, Zhongping Chen, Lihong Mu, Xiaojun Tang, Zhaohui Zhong, Juan Chen, Li Zhou

**Affiliations:** ^1^Department of Epidemiology, School of Public Health and Management, Chongqing Medical University, Chongqing, China; ^2^The People's Hospital of Jiulongpo District, Chongqing, China; ^3^The Key Laboratory of Molecular Biology of Infectious Diseases Designated by the Chinese Ministry of Education, Institute for Viral Hepatitis, Department of Infectious Diseases, The Second Affiliated Hospital, Chongqing Medical University, Chongqing, China

## Abstract

Hepatitis B virus (HBV) infection is a challenging public health problem in China and worldwide. Mother-to-child transmission is one of the main transmission routes of HBV in highly endemic regions. However, the mechanisms of HBV perinatal transmission in children have not been clearly defined. The aim of this study was to demonstrate the association between single-nucleotide polymorphisms (SNPs) in IFN-*γ* signaling pathway and HBV infection or breakthrough infection in children. Two hundred and seventy-four HBV-infected children defined as test positive for hepatitis B surface antigen (HBsAg) and 353 controls defined as negative for HBsAg in China were recruited from October 2013 to May 2015. SNPs in IFN-*γ* signaling pathway including IFNG, IFNGR1, IFNGR2, and IL12B were genotyped. Rs2234711 in IFNGR1 was significantly associated with HBV infection in children (OR = 0.641, 95% CI: 0.450–0.913). In addition, rs2234711 was also significantly associated with HBV breakthrough infection in children born to HBsAg-positive mothers (OR = 0.452, 95% CI: 0.205–0.998). Our study confirmed that genetic variants in IFN-*γ* signaling pathway have significant associations with HBV infection, especially with HBV breakthrough in children. This study provides insight into HBV infection in children and could be used to help design effective strategies for reducing immunoprophylaxis failure.

## 1. Background

Hepatitis B virus (HBV) infection is a major cause of acute and chronic liver disease, accounting for high morbidity and mortality of liver-associated disease worldwide [1]. Globally, approximately 2 billion people have serological evidence of HBV infection, leading to development of chronic infection in an estimated 257 million people and 887000 deaths in 2015 [2]. The initial age of HBV exposure is strongly associated with the possibility of developing chronic HBV infection (CHB). When infants are infected with HBV within 1 year after birth, 80–90% of them develop persistent infection. Before 6 years of age, 30–50% of infected children develop chronic HBV infection. For older children and adults infected with HBV, a lower rate (5–10%) of chronicity is observed [3]. A quarter of patients with CHB will progressively develop cirrhosis, liver failure, and/or hepatocellular carcinoma [4].

Vertical transmission is the main route of HBV transmission and contributes significantly to chronic HBV infection [5]. The burden of HBV infection in China is still the highest in the world, with one-third of CHB patient worldwide residing in China [2]. In China, an estimated 2 million HBV-infected women give birth every year, and 80–90% of these babies may develop chronic hepatitis B without intervention [6, 7]. Most of the vertical transmission of HBV occurs during birth or perinatal period [8]. Hepatitis B (HB) vaccine has been accessible since the early 1980s. To increase the coverage rate of HB vaccine, the government of China has vaccinated all infants with recombinant HB vaccine free of charge since 2002. Because the newborns have inoculated hepatitis B immunoglobulin (HBIG) and HBV vaccine with a three-dose series on a 0-, 1-, and 6-month schedule soon after birth, the rate of mother-to-infant transmission has been dramatically decreased [9, 10]. However, mother-to-infant transmission of HBV still fails to be completely prevented [11]. It was reported that nearly 10% of the children born to HBsAg and hepatitis B e antigen (HBeAg)-positive mothers did not achieve protective antibody levels and ultimately develop chronic hepatitis B (CHB) infection [12].

HBV infection is generally attributed to immunological factors, viral factors, environmental factors, and host genetic factors. A number of studies have identified the associations between polymorphisms in candidate genes and the outcomes of the HBV-infected disease [13, 14]. Interferon (IFN) family is a group of representative cytokines that elicit host innate immune responses against viral infections. Among three categories of interferon, type I (*α*, *β*, *ω* and *τ*), type II (*γ*), and type III (*λ*), interferon gamma (IFNG) has been identified as an important modulator of immune-related genes, including interferon gamma receptors (IFNGRs) [15, 16]. IFNGRs are required for IFNG to exert their biological functions and therefore play a very important role in IFN-*γ* signaling pathway [17, 18]. IFNGRs have two subunits, IFNGR1 and IFNGR2. IL12B is a potent IFN-*γ* inducing cytokine secreted by phagocytes and dendritic cells. Mutations in IL12B have been suggested to result in impaired IFN-*γ* production [19]. Some studies on the IFN-*γ* signaling pathway have examined the relationship between genetic variation and HBV infection. For example, IFN-*γ* + 874 and IFNGR1 (−56 and −611) have been identified as candidate genetic markers for determining susceptibility to the development of chronic hepatitis B in adults [20]. It was also reported that IL12B promoter heterozygosity (L/S) was associated with nonresponsiveness to HBV vaccination [21].

However, whether the genetic variation of IFN-*γ* signaling pathway gene affects the outcome of HBV infection and breakthrough infection in children still remains unknown. In this study, we designed a case-control study to explore the association between single-nucleotide polymorphisms (SNPs) in IFN-*γ* signaling pathway genes and HBV infection or breakthrough infection in children.

## 2. Materials and Methods

### 2.1. Study Participants

This study was designed as an analytical observational case-control study consisting of 274 cases (HBsAg positive) and 353 controls (HBsAg negative). All participants were unrelated ethnic Chinese between the ages of 6 months and 12 years, and consecutively recruited from October 2013 and May 2015 at the hospital of Chongqing medical university and health center for women and children in Chongqing. The study design has been described elsewhere [22]. The required sample size was calculated by QUANTO software [23]. Diseased children were enrolled if they were HBsAg positive for over 6 months, regardless of their alanine aminotransferase (ALT) levels, HBV DNA levels, or HBeAg status. Control children were recruited if they were HBsAg negative and had normal serum ALT levels. Subjects with other liver diseases or autoimmune diseases were excluded. Blood samples from children and their mothers (taken from arm venipuncture or scalp vein) were collected into tubes without any anticoagulant and stored at −80°C for further experimentation. Blood cells and sera were separated. Written informed consent was obtained from all parents or legal guardians of children involved in the study. This study was approved by the Ethics in Research Committee of Chongqing Medical University in Chongqing, China.

### 2.2. Target Genes and SNP Selection

A total of 4 genes belonging to the IFN-*γ* signaling pathway were selected as target genes. These genes included receptors for IFNs (IFNGR1, IFNGR2, and IL12B) and ligands for IFN receptors (IFNG). A total of 5 tagging SNPs located in the 4 genes were selected from the genotyped SNPs in the Chinese Han population of the Hap-Map project (the Phase II database) using the pairwise tagging method in Haploview 4.2 and further selected according to the literature. The selected SNPs met the following inclusion criteria: (1) minor allele frequency (MAF) ≥0.1 in Chinese Han population and (2) Hardy–Weinberg equilibrium (HWE) test with *P* value ≥ 0.05. These were IFNGR1 rs3799488 and rs2234711, IFNGR2 rs1059293, IL12B rs3212227, and IFNG rs1861494. The selected 5 SNPs have been reported to be associated with adult CHB or other diseases related to immune regulation. The basic information of these SNPs is demonstrated in Table 1.

### 2.3. Serological Assays

HBV markers (HBsAg, anti-HBs, HBeAg, anti-HBe, and anti-HBc) were detected by enzyme-linked immunosorbent assay (ELISA) in this study. Diagnostic kits were purchased from Shanghai Kehua Bioengineering Technology Company, Limited (Shanghai, China).

### 2.4. DNA Extraction

Human genomic DNA was isolated from EDTA noncoagulated blood samples using BioTeke DP6101 kit (BiotTeke corporation, Beijing, China). The DNA concentration was measured by Nanodrop2000c spectrophotometer (Thermo Scientific, DE). The eluted DNA was stored at −80°C for future experimentation.

### 2.5. SNP Genotyping

SNP genotyping was performed using the mass array time-of-flight mass spectrometer (Sequenom Company, USA) technique. Polymerase chain reaction (PCR) and extension primers were designed by the Mass ARRAY Assay Design 3.1 software (Sequenom Company, USA). The genotyping procedures were performed using the manufacturer's iPLEX Application Guide (Sequenom Company, USA). The PCR program for amplification conditions was 94°C, 15 min; 45 cycles × (94°C, 20 s; 56°C, 30 s; 72°C, 1 min); 72°C, 5 min. All the genotyping reactions were performed in 384-well plates, and each plate included four randomly selected duplicates and 6 negative controls; double distilled water was used as the negative controls. The average concordance rate of the genotype was 99.5%.

### 2.6. Analysis of Gene-Gene Interaction

In this study, the nonparametric multifactor dimensionality reduction (MDR; version 3.0.2) and generalized multifactor dimensionality reduction (GMDR; version 0.7) were used to test the potential interactions (one to six way combinations) among SNPs. The MDR was used to carry out the combination of cross-validation and permutation testing. The significance of permutation testing was determined using the GMDR. *P* value < 0.05 was considered significant.

### 2.7. Statistical Analysis

All data were double inputted in the computer using Epidata 3.02 software. SNPStats (http://bioinfo.iconcologia.net/snpstats/start.htm) and SPSS 21.0 (SPSS Inc., Chicago, IL, USA) were used to obtain odds ratios (ORs), 95% confidence intervals (CIs), and *P* values. The Hardy–Weinberg equilibrium of each SNP was used to test the deviation of genotype distribution using SNPStats. The SHEsis online platform was used to calculate the haplotype frequencies [24]. Multiple logistic regression models were applied in the analysis of the association of genotypes and HBV infection. Each component of the model was a codominant model (major allele homozygotes vs. heterozygotes vs. minor allele homozygotes), dominant model (major allele homozygotes vs. heterozygotes + minor allele homozygotes), recessive model (major allele homozygotes vs. minor allele homozygotes), overdominant model (major allele homozygotes + heterozygotes vs. minor allele homozygotes), and log-additive model. In the statistical analysis, *P* < 0.05 was considered significant.

## 3. Results

### 3.1. Patients Characteristics

Baseline characteristics of the participants have been described in our previous study [25]. Briefly, this study included 274 HBsAg-positive children (case group) with a mean age of 6.16 years (SD 4.02) and 353 healthy children (control group) with a mean age of 2.53 years (SD 1.49). The characteristic of sex demonstrated significant differences between the two groups (male/female: 180/94 in case group, 182/171 in control group, *P*=0.004). A total of 172 children born to HBsAg-positive mothers thus had been administrated with HBIG within 24 h after birth and HB vaccine according to the program (at 0, 1, and 6 months) in our study. Of these children, 40 participants were infected with HBV (40/172, 23.25%) and defined as HBV breakthrough infection children.

### 3.2. Association between SNPs and HBV Infection Risk in Children

The genotype distributions of five SNPs were all in the Hardy–Weinberg equilibrium in the controls. As represented in Table 2, SNP rs2234711 in IFNGR1 was associated with HBV infection after adjusting for sex and age. The rs2234711 AG genotype was found as a protective factor for HBV infection in children (OR = 0.641, 95% CI: 0.450–0.913).

We conducted genetic model analyses among the 5 SNPs, and the results are shown in Table 3. Four genetic models were applied in this study. In the dominant model, individuals carrying genotype GG at rs2234711 showed a decreased CHB risk with OR of 0.027 (95% CI = 0.491–0.957, *P*=0.027). In the codominant model, the rs2234711 genotype GA was found to be significantly different between cases and controls (OR = 0.643, 95% CI = 0.452–0.914, *P*=0.014). In the overdominant model, the genotype GA in rs2234711 was still found to be a protective factor for HBV infection in children (OR = 0.671, 95% CI = 0.485–0.928, *P*=0.016).

### 3.3. Association between SNPs and Risk of HBV Breakthrough Infection in Children Born to HBsAg (+) Mothers

We further studied the association between the 5 SNPs and HBV breakthrough infection in children who were born to HBsAg-positive mothers and had been vaccinated with HBIG and HB vaccine according to the standard procedure. As shown in Table 4, without adjustment for covariants, the genotype AG at rs2234711 in IFNGR1 was identified to be significantly associated with breakthrough infection in children whose mothers were HBsAg positive (OR = 0.452, 95% CI: 0.205–0.998, *P*=0.049). After adjusting for age and sex, it was found that there was a borderline association between rs2234711 genotype AG and HBV breakthrough infection in children, but it was not statistically significant (*P*=0.060, OR = 0.464, 95% CI: 0.208–1.033).

### 3.4. Gene-Gene Interaction in IFN-*γ* Signaling Pathway

The results of GMDR model analysis for the entire case-control data are shown in Table 5, which showed that the best model was the one-factor model, but there were no significant interactions with polymorphisms in the four genes in IFN-*γ* signaling pathway.

### 3.5. Haplotype Analyses

The SHEsis online program was applied to calculate the degree of linkage disequilibrium of the two IFNGR1 SNPs and haplotype frequencies in this study. Pairwise LD analysis between the two SNPs was performed, and the D′ value between rs2234711 and rs3799488 was equal to 1.0 (*r*^2^ = 0.262). According to the sequence of rs2234711-rs3799488, the AT, GC, and GT haplotypes, with the smallest haplotype frequency >0.03, were included in the study as common haplotypes. The results showed that there were no significant differences in haplotypes frequency distribution between cases and controls (*P* > 0.05), as listed in Table 6.

## 4. Discussion

Chronic HBV infection remains a serious global health problem [26, 27]. It was reported that host genetic factors played an important role in the pathogenesis of HBV infection, and the polymorphisms of cytokine genes were strongly associated with the susceptibility to HBV infection in the general population [28, 29].There is enough evidence that genetic variants of IFN-*γ* signaling pathway genes play important roles in the HBV infection in adults [30–32]. But there is no study demonstrating the association between gene polymorphisms of IFN-*γ* signaling pathway and HBV infection in children. In the present study, we evaluated whether the genetic variations in genes of IFN-*γ* signaling pathway including IFNGR1 (rs3799488 and rs2234711), IFNGR2 (rs1059293), IL12B (rs3212227), and IFNG (rs1861494) could have an impact on the susceptibility to HBV infection in children. The result showed that only rs2234711 AG in IFNGR1 was significantly associated with HBV infection in children.

IFNGR1 gene polymorphisms were correlated with a lot of diseases [33–35]. Zhou et al. demonstrated that IFNGR1-56C/T SNP affected the clinical outcome of HBV infection [30]. In another study, IFNGR-1 (−56) was suggested as a candidate gene marker for determining susceptibility to the development of chronic hepatitis B [20]. Our study revealed that rs2234711 AG in IFNGR1 was significantly associated with HBV infection in children. Additionally, the result by genetic model analysis showed that variant rs2234711 (GA + AA/GG, GG/AG, and GG + AA/GA) was still significantly associated with HBV infection, suggesting that rs2234711 was strongly related to HBV infection in children. However, Cheong et al. demonstrated that there was no association between −56C/T SNP and persistent HBV infection in Korean population [36]. This discrepancy may come from the differential genetic makeup in the two populations. More importantly, our subjects were children, whose immune system was not yet fully developed; thus, the different immune mechanisms to address hepatitis virus may be one of the reasons.

The location of SNP rs2234711 is at the 5′-UTR of IFNGR1, which indicates that this SNP is likely to be a causal variant involved in the regulation of the promoter activity. HaploReg data indicate that rs2234711 is located in a region with promoter histone marks in 24 tissues, DNase hypersensitivity site in 53 tissues, and alters 4 in silico transcription factor binding sites. It also highlights that rs2234711 is located in a gene regulatory hotspot. Genotype-Tissue Expression (GTEx) project database shows that rs2234711 genotype is significantly associated with IFNGR1 expression level in whole blood (*P*=3.0 × 10^−7^). Therefore, the risk allele of rs2234711 is demonstrated to reduce promoter activity and cis-regulate IFNGR1 expression. In the IFN-*γ* signaling pathway, IFN-*γ* binds to the transmembrane molecular IFNGR1 [37]. On ligand binding, phosphorylation of STAT1 is activated. Acting as a transcriptional activator, phosphorylated STAT1 induces downstream immunomodulatory gene expression including IL-12 [35]. Numerous studies have indicated that STAT1 and IL-12 play a key role in mediating defensive mechanism against HBV [38–40]. An experimental research also indicated that IFN-*γ* receptor gene defect was conducive to HBV infection [41]. As stated above, rs2234711 is shown to be the causal variant of HBV infection depending on the IFN-*γ* signaling pathway.

On the basis of unconditional logistic regression analysis with adjustment for age and sex, no statistically significant association with susceptibility to HBV infection and breakthrough infection in children was observed with other 4 SNPs (rs3799488 in IFNGR1, rs1059293 in IFNGR2, rs3212227 in IL12B, and rs1861494 in IFNG). However, a significant association between polymorphisms in rs3799488, rs1059293, and rs3212227 and the risk of chronic HBV infection was confirmed by He et al. [31]. This discrepancy may also be attributed to the difference in samples involved in the two studies. IFN-*γ* (IFNG) plays a crucial role in the immune system and seems to be involved in the clearance of HBV infection [17]. A previous study demonstrated that polymorphisms in IFNG affected the IFN-*γ* expression level, which contributed to different clinical outcomes of HBV infection [42]. Another meta-analysis showed that IFN-*γ* rs2430561T>A was an important factor that promoted the pathogenesis of HBV infection [43]. Although the variant rs1861494 in IFN-*γ* had been reported to be associated with many other disease [44], only one research involving subjects of Chinese ethnic minority suggested that genotype CC at rs1861494 conferred an increased risk for HBV susceptibility in both Dai and Hani minorities [45]. In our study, there was no significant association between rs1861494 genotype and HBV infection in children, which was consistent with He et al.‘s research.

In China, the administration of HBIG and HB vaccine to infants who are born to HBsAg-positive mothers has been the most effective preventive measure against vertical transmission of HBV [46, 47]. However, the breakthrough infections in children do occur [48]. Previous studies have suggested that host genetic variants were responsible for the failure of immunoprophylaxis [49]. Moreover, our previous study demonstrated that rs3917267 genotype AA in IL1R1 gene was a risk factor for HBV breakthrough infection in children [25]. In the present study, we further explored the association between the SNPs in genes of IFN-*γ* signaling pathway and HBV breakthrough infection. The result indicated that, without adjustment for age and sex, only the rs2234711 genotype AG showed a borderline association with HBV breakthrough infection (OR = 0.452, 95% CI = 0.205–0.998, *P*=0.049), but none of these SNPs was significantly associated with HBV breakthrough infection in children after adjusting for age and sex.

Some limitations should be considered in the present study. Firstly, the sample size was relatively small, which would cause the limited power for statistical analysis. Larger studies, especially through multicenter collaboration, will be needed to fully validate the significance of these findings. Secondly, the information on HBV DNA genotypes and virus load of both children and mothers was lacking. There are more than one HBV genotype distributed worldwide, and the main HBV categories in China are genotype B and genotype C, which might also affect the susceptibility to HBV infection. Thirdly, some baseline characteristics such as feeding pattern and delivery mode were lacking, which limited the performance of confirming the transmission route that actually caused infection. Therefore, the next step is to expand the sample size and complete the basic information of the subjects to test and verify the findings of this study.

## 5. Conclusion

The 5 genetic variants (rs3799488, rs2234711, rs1059293, rs3212227, and rs1861494) in IFN-*γ* signaling pathway genes (including IFNGR1, IFNGR2, IL12B, and IFNG) were investigated in this study. Only rs2234711 in IFNGR1 has significant associations with HBV infection and breakthrough in children. However, the role of IFN-*γ* signaling pathway gene polymorphisms in HBV breakthrough infection needs further investigation based on a large population and more genes or SNPs in this pathway, and further study in functional significance is also required.

## Figures and Tables

**Table 1:** Basic information of the selected SNP loci in this study.

SNP	Chr	Chr. position	Alleles	Gene	SNP property	MAF (HapMap-HCB)	MAF (present study)
rs3212227	5	159315942	G/T	IL12B	3′-UTR	0.430	0.468
rs3799488	6	137198643	C/T	IFNGR1	Intron variant	0.190	0.290
rs2234711	6	137219383	A/G	IFNGR1	5′-UTR	0.500	0.388
rs1861494	12	68157629	T/C	IFNG	Intron variant	0.333	0.326
rs1059293	21	33437386	C/T	IFNGR2	3′-UTR	0.128	0.126

**Table 2:** Association analysis of IFN pathway gene polymorphisms in children with HBV infection.

Gene	SNP	Genotype	Case (%)	Controls (%)	Crude OR (95% CI)	*P* value	Adjust OR (95% CI)^*∗*^	*P* value^*∗*^
IFNGR1	rs3799488	TT	131 (48.0)	177 (52.1)	1		1	
		CT	119 (43.6)	136 (40)	1.182 (0.847–1.651)	0.326	1.213 (0.865–1.701)	0.262
		CC	23 (8.4)	27 (7.9)	1.151 (0.631–2.098)	0.646	1.137 (0.620–2.084)	0.679
	rs2234711	GG	113 (41.9)	113 (33.1)	1		1	
		AG	116 (43.0)	180 (52.8)	0.644 (0.454–0.914)	**0.014**	0.641 (0.450–0.913)	**0.014**
		AA	41 (15.2)	48 (14.1)	0.854 (0.522–1.397)	0.53	0.851 (0.518–1.399)	0.525

IFNGR2	rs1059293	TT	205 (74.8)	264 (77.9)	1		1	
		CT	65 (23.7)	68 (20.1)	1.231 (0.837–1.811)	0.291	1.209 (0.819–1.785)	0.339
		CC	4 (1.5)	7 (2.0)	0.736 (0.213–2.548)	0.628	0.825 (0.235–2.893)	0.764

IL12B	rs3212227	TT	77 (28.3)	90 (26.5)	1		1	
		GT	138 (50.7)	179 (52,6)	0.901 (0.618–1.313)	0.588	0.911 (0.623–1.332)	0.629
		GG	57 (21.0)	71 (20.9)	0.938 (0.591–1.490)	0.788	0.917 (0.575–1.463)	0.717

IFNG	rs1861494	TT	129 (47.1)	156 (45.1)	1		1	
		TC	108 (39.4)	158 (45.7)	0.827 (0.589–1.159)	0.27	0.818 (0.581–1.151)	0.249
		CC	37 (13.5)	32 (9.2)	1.398 (0.825–2.370)	0.213	1.399 (0.821–2.386)	0.217

^*∗*^Adjusting for sex and age.

**Table 3:** Associated between SNPs and HBV infection in children using genetic models.

Gene	SNP	Model	Genotype	Crude OR (95% CI)	*P* value	Adjusted OR (95% CI)^*∗*^	*P* value^*∗*^
IFNGR1	rs3799488	Dominant	TC + CC/TT	1.177 (0.856–1.619)	0.316	1.200 (0.870–1.657)	0.267
		Recessive	TT + TC/CC	1.067 (0.597–1.906)	0.828	1.041 (0.579–1.872)	0.893
		Codominant	TT/CT	1.182 (0.847–1.651)	0.326	1.212 (0.864–1.698)	0.265
			TT/CC	1.151 (0.631–2.098)	0.646	1.132 (0.613–2.090)	0.692
		Overdominant	TT + CC/TC	1.159 (0.839–1.601)	0.370	1.192 (0.859–1.653)	0.293
	rs2234711	Dominant	GA + AA/GG	0.689 (0.495–0.958)	**0.027**	0.685 (0.491–0.957)	**0.027**
		Recessive	GG + GA/AA	1.093 (0.696–1.716)	0.700	1.092 (0.693–1.723)	0.704
		Codominant	GG/AG	0.644 (0.454–0.914)	**0.014**	0.643 (0.452–0.914)	**0.014**
			GG/AA	0.854 (0.522–1.397)	0.530	0.850 (0.516–1.400)	0.523
		Overdominant	GG + AA/GA	0.674 (0.489–0.929)	**0.016**	0.671 (0.485–0.928)	**0.016**

IL12B	rs32172227	Dominant	TG + GG/TT	0.912 (0.638–1.303)	0.612	0.913 (0.636–1.309)	0.619
		Recessive	TT + TG/GG	1.004 (0.679–1.486)	0.982	0.975 (0.656–1.449)	0.901
		Codominant	TT/GT	0.901 (0.618–1.313)	0.588	0.911 (0.623–1.333)	0.631
			TT/GG	0.938 (0.591–1.490)	0.788	0.916 (0.574–1.463)	0.713
		Overdominant	TT + GG/TG	0.926 (0.673–1.274)	0.638	0.945 (0.685–1.305)	0.732

IFNGR2	rs1059293	Dominant	TC + CC/TT	1.185 (0.815–1.723)	0.375	1.176 (0.806–1.716)	0.401
		Recessive	TT + TC/CC	0.703 (0.204–2.425)	0.577	0.792 (0.227–2.768)	0.715
		Codominant	TT/CT	1.231 (0.837–1.811)	0.291	1.209 (0.819–1.785)	0.338
			TT/CC	0.736 (0.213–2.548)	0.628	0.816 (0.233–2.855)	0.750
		Overdominant	TT + CC/TC	1.239 (0.843–1.821)	0.274	1.214 (0.823–1.791)	0.327

IFNG	rs1861494	Dominant	TC + CC/TT	0.923 (0.672–1.268)	0.621	0.915 (0.663–1.262)	0.589
		Recessive	TT + TC/CC	1.532 (0.927–2.532)	0.096	1.541 (0.927–2.562)	0.095
		Codominant	TT/TC	0.827 (0.589–1.159)	0.270	0.816 (0.579–1.150)	0.246
			TT/CC	1.398 (0.825–2.370)	0.213	1.397 (0.821–2.377)	0.217
		Overdominant	TT + CC/TC	0.774 (0.561–1.068)	0.119	0.766 (0.553–1.060)	0.108

^*∗*^Adjusting for sex and age.

**Table 4:** Association between SNPs and risk of HBV breakthrough infection in children born to HBsAg (+) mothers.

Gene	SNP	Genotype	Case	Controls	Crude OR (95% CI)	*P* value	Adjust OR (95% CI)^*∗*^	*P* value^*∗*^
IFNGR1	rs3799488	TT	19 (47.5)	67 (55.4)	1		1	
		CT	16 (40.0)	49 (40.5)	1.151 (0.538–2.463)	0.716	1.112 (0.514–2.403)	0.787
		CC	5 (12.5)	5 (4.1)	3.526 (0.923–13.47)	0.065	3.492 (0.892–13.669)	0.073
	rs2234711	GG	18 (45.0)	38 (31.1)	1		1	
		AG	15 (37.5)	70 (57.4)	0.452 (0.205–0.998)	**0.049**	0.464 (0.208–1.033)	0.060
		AA	7 (17.5)	14 (11.5)	1.056 (0.363–3.067)	0.921	1.034 (0.351–3.050)	0.951

IFNGR2	rs1059293	TT	30 (75.0)	90 (75.0)	1		1	
		CT	8 (20.0)	28 (23.3)	0.857 (0.353–2.083)	0.734	0.915 (0.372–2.251)	0.846
		CC	2 (5.0)	2 (1.7)	3.00 (0.405–22.235)	0.282	3.332 (0.432–25.723)	0.248

IL12B	rs3212227	TT	12 (30.0)	30 (24.6)	1			
		GT	18 (45.0)	69 (56.6)	0.652 (0.280–1.521)	0.323	0.673 (0.285–1.585)	0.365
		GG	10 (25.0)	23 (18.9)	1.087 (0.400–2.954)	0.870	1.178 (0.426–3.257)	0.752

IFNG	rs1861494	TT	20 (50.0)	56 (43.8)	1		1	
		TC	14 (35.0)	61 (47.7)	0.643 (0.297–1.393)	0.262	0.680 (0.310–1.489)	0.335
		CC	6 (15.0)	11 (8.6)	1.527 (0.499–4.672)	0.458	1.466 (0.471–4.564)	0.509

^*∗*^Adjusting for sex and age.

**Table 5:** The models to predict chronic HBV infection in children by GMDR.

Factor	Model	Training bal.acc.	Testing bal.acc.	Sign test (P)	CV consistency
1	rs2234711	0.5523	0.5523	**9 (0.0107)**	10/10
2	rs2234711, rs1861484	0.5714	0.5035	5 (0.6230)	4/10
3	rs2234711, rs1861484, rs3212227	0.5999	0.4557	2 (0.9893)	6/10
4	rs2234711, rs1861484, rs3212227, rs3799488	0.6415	0.4786	5 (0.6230)	5/10
5	rs2234711, rs1861484, rs3212227, rs3799488, rs1059293	0.6804	0.5033	7 (0.1719)	10/10

**Table 6:** Associations between the IFNGR1 haplotypes and chronc HBV infection in children.

Haplotype^*∗*^	Cases (%)	Controls (%)	OR (95% CI)	*P* value
AT	198 (36.7)	270 (40.4)	1.000	–
GC	163 (30.2)	186 (27.8)	1.195 (0.904–1.580)	0.211
GT	179 (33.1)	212 (31.7)	1.151 (0.878–1.509)	0.307

^*∗*^The sequence of SNPs is rs2234711 and rs3799488.

## Data Availability

The data used to support the findings of this study are available from the corresponding author upon request.
